# Effect of intraspecific seed trait variation on the germination of eight tropical dry forest species

**DOI:** 10.1007/s00114-024-01898-5

**Published:** 2024-03-22

**Authors:** Natalia Villa-Rivera, Jeiner Castellanos-Barliza, Ariadna Mondragón-Botero, Willinton Barranco-Pérez

**Affiliations:** 1 Grupo de Investigación en Restauración Ecosistémica y Ecología Urbana, Facultad de Ciencias Básicas, Universidad del Magdalena, Carrera 32#22-08, Santa Marta D.T.C.H., 470002 Magdalena, Colombia; 2 Department of Plant and Microbial Biology, University of Minnesota, St. Paul, MN 55108 USA

**Keywords:** Nutrients, Functional traits, Seed mass, Seed volume, Germination speed

## Abstract

Functional traits can have intraspecific and interspecific variations essential in the structure and dynamics of natural communities. These traits may have implications in the germination and seedling establishment phases in seeds. The objective of this study was to evaluate the effect of variations in mass, volume, and nutrient content (C, N, and P) on the germination of eight species representative of the tropical dry forest (TDF). Our results showed that seed size, both in terms of mass and volume, did not predict germination rates or percentages, nor were they related to nutrient content. In contrast, N content was the most important trait in the germination phase. Larger seeds did not germinate more or faster, but they could offer better resistance against desiccation, since they had higher *C*/*N* ratios in their tissues, a characteristic of orthodox seeds. The species *A. guachapele*, *B. arborea*, *H. crepitans*, and *V. tortuosa* presented a high biological potential in terms of their regeneration capacity, particularly, because the characteristics of their seeds, as well as the nutrient content, revealed consistent implications in their reproductive success, promoting high germination percentages in less time. In general, the results obtained in this study provide basic knowledge for future research, offering starting points for further exploration of species-specific adaptations and how they may be affected by the environment.

## Introduction

Functional traits represent morphological, physiological, structural, and phenological adaptations that species have developed to survive climatic variations in their inhabited ecosystems (Rodríguez-Alarcón et al. 2020). These traits can show intraspecific and interspecific variations essential for the structure and dynamics of natural communities (Salgado-Negret 2016; Faccion et al. 2021). For example, in Tropical Dry Forest (TDF), species have developed different strategies to cope with prolonged periods of drought. Some may be deciduous or modify their leaves into thorns to reduce evapotranspiration, while others present adaptations in their stems and roots to conserve water (Pizano and García 2014; Chaturvedi et al. 2021). Likewise, phenological patterns of leaf production, flowering, and fruiting can synchronize with rainy periods, increasing the probability of reproductive success (Cárdenas-Henao et al. 2015; Suresh and Nanda 2021). Seed morphology traits observed in TDF favor seed dispersal by wind or animals, and secondary dormancy in the soil until conditions are suitable for germination (Vargas 2012; Pérez-Martínez et al. 2014). Similarly, morphological traits such as seed size (mass or volume) are associated with seed germination rates and seedling viability and establishment in these ecosystems (Moles et al. 2005; Khurana et al. 2006; Romero-Saritama and Pérez-Ruiz 2016; Romero-Saritama and Castillo 2022). It has been observed that larger and heavier seeds with high water content and the presence of oleosinic proteins are more likely to survive dehydration (Plaza and Magnitskiy 2007). However, despite these findings, more empirical evidence is needed to support the relationships of these morphological traits in TDF.

General patterns in TDF have revealed that traits such as seed size are important for increasing the probability of seedling establishment (Khurana et al. 2006; Romero-Saritama and Castillo 2022). For example, larger seeds favor higher seedling performance on sites with low resource availability, whereas small seeds are produced in greater quantity and thus favor species recruitment to potentially less stressful sites (Pinho et al. 2019). Most species in TDF are considered orthodox (89%), which are characterized by tolerating desiccation, presenting small sizes (10 ± 8 mm long and 6.2 ± 4 mm wide) and not exceeding one gram in weight (Romero-Saritama and Pérez Ruiz 2016; Romero-Saritama and Castillo 2022). However, there are some notable exceptions in some species, such as *Hura crepitans*, *Cavanillesia platanifolia*, *Geoffroea spinosa*, and *Tabebuia chrysantha*, which have larger seed sizes (18 mm long and 3 g in weight) and have high germination and survival rates (Romero-Saritama and Castillo 2022). It has been proposed that having medium or large seeds can be an advantage for plants under high water stress conditions, and having small seeds favors the reproductive success of plants under moderate water stress conditions. This is because the water requirements of a small seed are lower and it can germinate at low water levels. Large seeds have the ability to synchronize their germination with periods of rainfall, withstanding drought and providing seedlings with more resources for growth (Khurana et al. 2006). Additionally, traits such as nutrient content in seeds play an important role in the physiological processes that initiate germination, as these nutrient reserves stimulate metabolic processes that provide energy for embryonic development in plants (Milberg and Lamont 1997; Lamont and Groom 2013; Soriano et al. 2015). This phenomenon has been observed in certain species of the Fabaceae family, which have developed a symbiotic association with nitrogen (N)-fixing bacteria (De Bedout-Mora et al. 2022). In particular, a positive correlation has been observed between the presence of N-fixing nodules and increased nitrogen content in seed endosperm tissues, leading to a reduction in the time required for germination (Valencia-Díaz et al. 2015; Mathesius 2022). The effect of nutrient content on germination has been previously observed by Soriano et al. (2011) in 19 TDF species in northwestern Mexico. These results indicated that larger seeds tended to have a lower N concentration, showing a proportional increase in dry mass allocation to the seed coat. Thus, both traits were associated in this study with lower germination rates and stress persistence strategies, including shade tolerance in tropical trees.

Despite the importance of these strategies at the seed level for seedling establishment and survival, such traits have not been well studied functionally in different habitats, especially in the TDF. Therefore, such studies are critical to assess the permanence and sustainability of these dry ecosystems over time. Similarly, seed traits have received much less attention than foliar traits or root traits (Liu et al. 2022; Visscher et al. 2022). Therefore, it is essential to expand the knowledge of seed traits that influence seedling regeneration to implement adequate species selection strategies for the restoration of TDF in the future.

Consequently, we used seven functional traits of eight representative TDF species to evaluate the effect of variation in mass, volume, and nutrient content (C, N, and P) on ex situ seed germination. We hypothesize that, among the groups of species studied, those with greater mass and volume have high C, N, and P contents. Accordingly, high germination percentages and shorter germination times for these species are observed. On the contrary, seeds with lower mass and volume are associated with lower nutrient contents and longer germination times.

This study is expected to generate knowledge that can be used to create ex situ conservation and reproduction strategies for tree species and later be implemented in reforestation plans in the degraded TDF of the Colombian Caribbean region.

## Materials and methods

### Study area

**Fig. 1:**
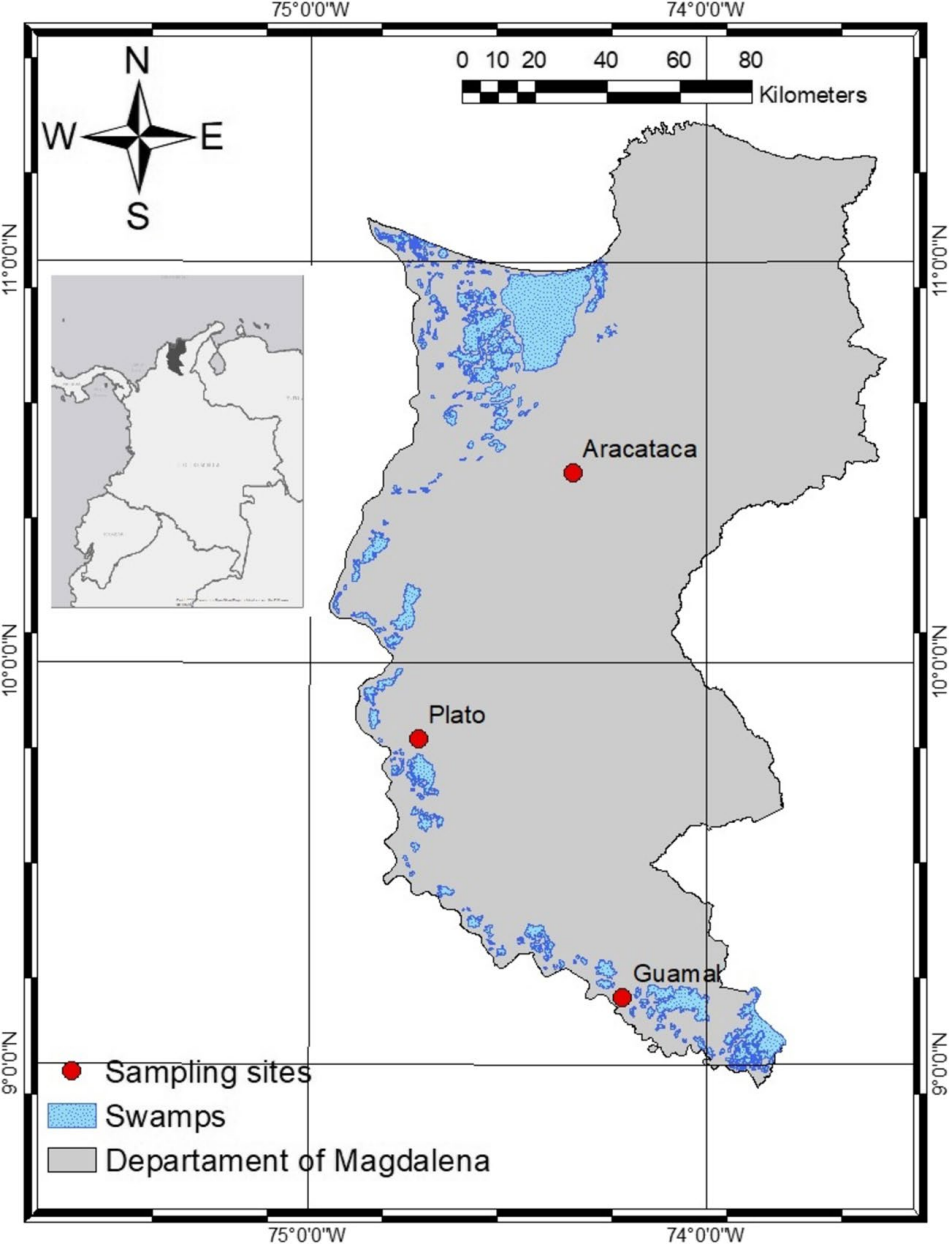
Geographical location of the three fragments of TDF in Magdalena, Colombia, where seeds were collected

For this study, seeds of native species were collected in three fragments of TDF in the department of Magdalena, Colombia (Fig. 1). These forests are characterized by long periods of drought that last between 3 and 8 months, with one or two periods of annual rainfall and an average precipitation between 250 and 2000 mm. The canopy can reach 10–12 m in height and is dominated by species of the Fabaceae, Anacardiaceae, and Malvaceae families, such as *Machaerium goudotii* Benth, *Pterocarpus acapulcensis* Rose, *Astronium graveolens* Jacq., and *Pseudobombax septenatum* (Jacq.) Dugand (Castellanos-Barliza et al. 2022; Londoño-Lemos et al. 2022).

### Selected species and seed collection

**Fig. 2:**
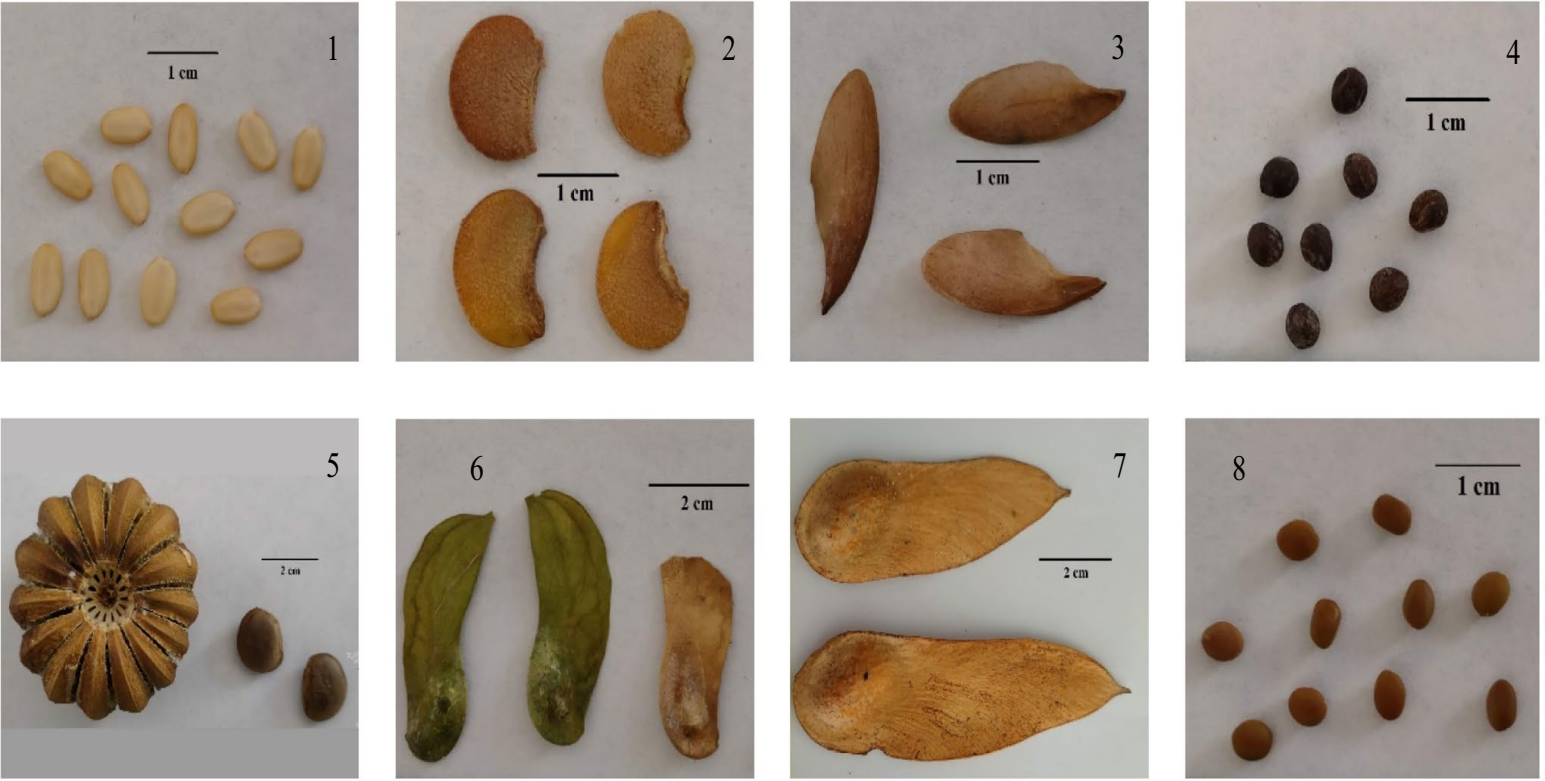
Seeds of the eight native TDF species studied: *A. guachapele* (1), *B. arborea* (2), *C. odorata* (3), *C. pentandra* (4), *H. crepitans* (5), *M. balsamum*, (6), *P. elegans* (7), and *V. tortuosa* (8)

A total of eight native species of the TDF in the Department of Magdalena were selected (Fig. 2): (1) *Ceiba pentandra* (L.) Gaertn. (Fabaceae), (2) *Albizia guachapele* (Kunth) Dugand. (Fabaceae), (3) *Cedrela odorata* L. (Meliaceae), (4) *Platypodium elegans* Vogel. (Fabaceae), (5) *Hura crepitans* L. (Euphorbiaceae), (6) *Myroxylon balsamum* (L.) Harms. (Fabaceae), (7) *Bulnesia arborea* (Jacq.) (Zygophyllaceae), and (8) *Vachellia tortuosa* (L.) Seigler y Ebinger. (Fabaceae). The composition and quantity of these species were selected considering their representativeness in the three forest fragments visited, as well as the availability of seeds during the collection period (April-March 2021).

Seeds were collected following the protocol for collecting, processing, and storing wild plant seeds proposed by Di Sacco et al. (2020). Approximately 300 seeds of each species were manually collected directly from the trees and deposited in paper bags. Since certain species, such as *C. pentandra*, *A. guachapele*, *C. odorata*, and *H. crepitans*, have dehiscent fruits, we collected ripe fruits characterized by their dry appearance and dark brown coloration. They were then taken to the laboratory where they were stored in semi-hermetic containers to protect them from humidity, air, light, and animals and insects to avoid damage and prevent a decrease in viability.

### Measurement of functional traits

Measurement of seed traits was performed following the standardized protocols (Pérez-Harguindeguy et al. 2013). Thus, 300 seeds per species were taken and the experimental trial was carried out with them. Particularly in the case of *M. balsamum* and *P. elegan* species, it was decided to measure the samaras, since it was difficult to extract the seeds without damaging them. Seed mass was calculated as dry weight (g). Therefore, three subsamples of seeds were taken from each species, which were subjected to the oven at a temperature of 65 °C until a constant dry weight was obtained. Seed volume (mm^3^) was calculated according to Eq. 1:

1 $$V=\pi /6\times L1\times L2\times L3$$

where *V* is the volume, *L*1 is the length, *L*2 is the width, and *L*3 is the thickness.

For determining C, N, and P content, subsamples of 5 to 50 seeds of each species were taken and ground to obtain a minimum of 10 g per species. The crushed seeds were packed in zip-lock bags and sent to the laboratory at the International Center for Tropical Agriculture in Palmira, Colombia. Carbon and nitrogen were determined by combustion in an Elemental Analyzer, and the phosphorus content was determined by atomic absorption and ultraviolet visible tests (Murphy and Riley 1962; Sleutel et al. 2007). The values were recorded as a percentage of the nutrient. Carbon–nitrogen (*C*/*N*) and nitrogen-phosphorus (*N*/*P*) ratios were subsequently calculated with these values.

### Germination tests

The collected seeds were examined for any defects and properly cleaned. Those with good phytosanitary status, i.e., those unaffected by fungi, insects, or morphological defects, were selected. Subsequently, these seeds were subjected to pre-germinative treatments to break dormancy (Abril-Saltos et al. 2017). Hard-coated seeds, such as those of *A. guachapele*, *H. crepitans*, *M. balsamum*, *P. elegans*, and *V. tortuosa*, were scarified and soaked in hot water for 2 h at a temperature up to 90 °C, while the rest of the seeds were only soaked for 2 h. Consequently, 300 seeds of each species were sown into bags a nursery at the Universidad del Magdalena, Colombia. The substrate for sowing was prepared with soil from one of the TDF sites and sand in a 3:1 ratio. Seeds were monitored to record germination parameters. The time at which the plumule emerged from the soil was defined as the germination criterion for this study. The germination percentage for each species was calculated as the number of germinated seeds divided by the total number of seeds multiplied by 100 (Eq. 2). Germination speed (GS) was calculated as the reciprocal of the mean germination time (AGT, Eqs. 3 and 4; Ranal and De Santana 2006; Soltani et al. 2015).

2 $$\mathrm{Germination}\;\mathrm{percentage}\;(\%)\;=\;\mathrm{No}.\;\mathrm{seeds}\;\mathrm{germinated}\;/\;\mathrm{No}.\;\mathrm{seeds}\;\mathrm{sown}\;\times\;100$$

3 $$\text{AGT}=\left(\sum\nolimits_{i=1}^k\left[n_it_i\right]\right)/\left(\sum\nolimits_{i=1}^k\left[n_i\right]\right)$$

where AGT (day) is average germination time, *T*_*i*_ is the time from the start of the experiment to the *i*-th observation in days, *n*_*i*_ is the number of seeds germinated at time *i*, and *k* is the last day of germination.

4 $${\text{GS}}=1/{\text{AGT}}$$

where GS (days^−1^) is the germination speed.

### Data analysis

Seed functional trait means and standard errors (mean ± SE) were calculated. To determine significant differences in functional traits among species, Univariate Analysis of Variances (ANOVA) were performed, followed by a Tukey’s test when significant differences were detected (*p* ≤ 0.05). A Kruskal–Wallis test followed by a multiple-contrast Bonferroni test was performed when the residuals did not fit a normal distribution. A principal component analysis (PCA) was performed to visualize the associations between traits, germination percentage, and germination speed. Spearman’s correlations were also performed to observe the type of relationships between the functional traits of the seeds and the studied germination parameters. In addition, multiple linear regression models were fitted to determine the most determinant (explanatory) variables of average speed and germination. The “Stepwise” method was applied to identify these variables, which consists of eliminating or adding explanatory variables step by step. To select the best definitive models, several fitting criteria were used, including Durbin-Watson (DW), Mean Squares Error (CME), Akaike Information Criterion (AIC), and Root Mean Square Error (RMSE). These procedures were performed with Statgraphics Centurion XVII (StatPoint Technologies, Inc.) and R version 4.2.1 (packages: factoextra, stats, olsrr, parameters; Hebbali 2020; Lüdecke et al. 2020; Kassambara and Mundt 2022; R Core Team 2022).

## Results

### Functional traits

Significant differences among species were observed in seed functional traits (Table 1, gl = 7, *p* = 0.0001). The species *P. elegans*, *H. crepitans*, and *M. balsamum* presented higher masses and volumes. In contrary, lower values were recorded in *C. pentandra*, *A. guachapele*, *C. odorata*, *B. arborea*, and *V. tortuosa*. Additionally, high N contents were observed in *A. guachapele*, *H. crepitans*, and *V. tortuosa*, representing more than 50% of that recorded for the total species. High *P* contents were observed for *P. elegans* and lower for *C. pentandra.*

**Table 1:** Mean value and standard error of functional seed traits in eight TDF tree species of Magdalena, Colombia

Species	Mass (g)	Volume (mm^3^)	C (%)	N (%)	P (%)	*C*/*N*	*N*/*P*
*C. pentandra*	0.01 ± 0.000f	51.53 ± 1.656 cd	40.40 ± 1.061a	2.84 ± 0.106de	1.01 ± 0.008a	14.23 ± 0.158ab	2.81 ± 0.085 g
*A. guachapele*	0.05 ± 0.001d	31.61 ± 0.891de	39.19 ± 2.998a	6.07 ± 0.019a	0.44 ± 0.004de	6.45 ± 0.473e	13.90 ± 0.180a
*C. odorata*	0.01 ± 0.000 g	90.97 ± 5.645bc	42.75 ± 1.467a	4.36 ± 0.189c	0.69 ± 0.026b	9.81 ± 0.089c	6.36 ± 0.037f
*P. elegans*	1.07 ± 0.018a	8252.82 ± 174.026a	42.25 ± 0.689a	3.18 ± 0.060d	0.25 ± 0.004f	13.20 ± 0.468b	12.75 ± 0.053b
*H. crepitans*	0.96 ± 0.007a	1278.14 ± 51.885ab	46.49 ± 1.775a	5.39 ± 0.133b	0.71 ± 0.002b	8.61 ± 0.117 cd	7.64 ± 0.163e
*M. balsamum*	0.16 ± 0.006b	1386.99 ± 44.179ab	43.16 ± 2.542a	2.66 ± 0.092e	0.40 ± 0.023e	16.30 ± 1.523a	6.69 ± 0.157f
*B. arborea*	0.13 ± 0.005c	118.73 ± 4.944b	42.36 ± 1.153a	4.11 ± 0.011c	0.48 ± 0.011 cd	10.30 ± 0.307c	8.58 ± 0.225d
*V. tortuosa*	0.04 ± 0.001e	20.55 ± 0.750e	42.03 ± 0.764a	5.77 ± 0.001ab	0.52 ± 0.004c	7.28 ± 0.134de	11.20 ± 0.081c

Different letters indicate statistically significant differences between species (*p* < 0.05)

*C* carbon, *N* nitrogen, *P* phosphorus, *C/N* carbon-to-nitrogen ratio, *N/P* nitrogen-to-phosphorus ratio

### Seed germination

**Fig. 3:**
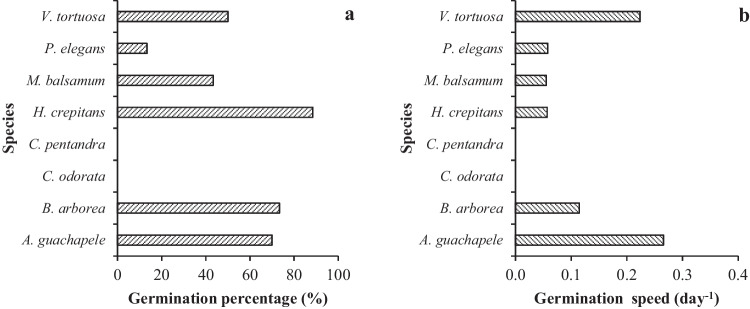
Germination percentage (**a**) and germination speed (**b**) of seeds in eight tree species of the TDF of Magdalena, Colombia

The first germination events were observed three days after sowing and were completed after 22 days. The species *V. tortuosa* and *A. guachapele* had high germination percentages and speeds. *B. arborea* and *H. crepitans* showed high germination but a low germination speed (Fig. 3a, b). Overall, *P. elegans* showed the lowest germination percentage and germination speed in the study. Conversely, seeds of *C. pentandra* and *C. odorata* species did not germinate.

### Effect of traits on seed germination

**Fig. 4:**
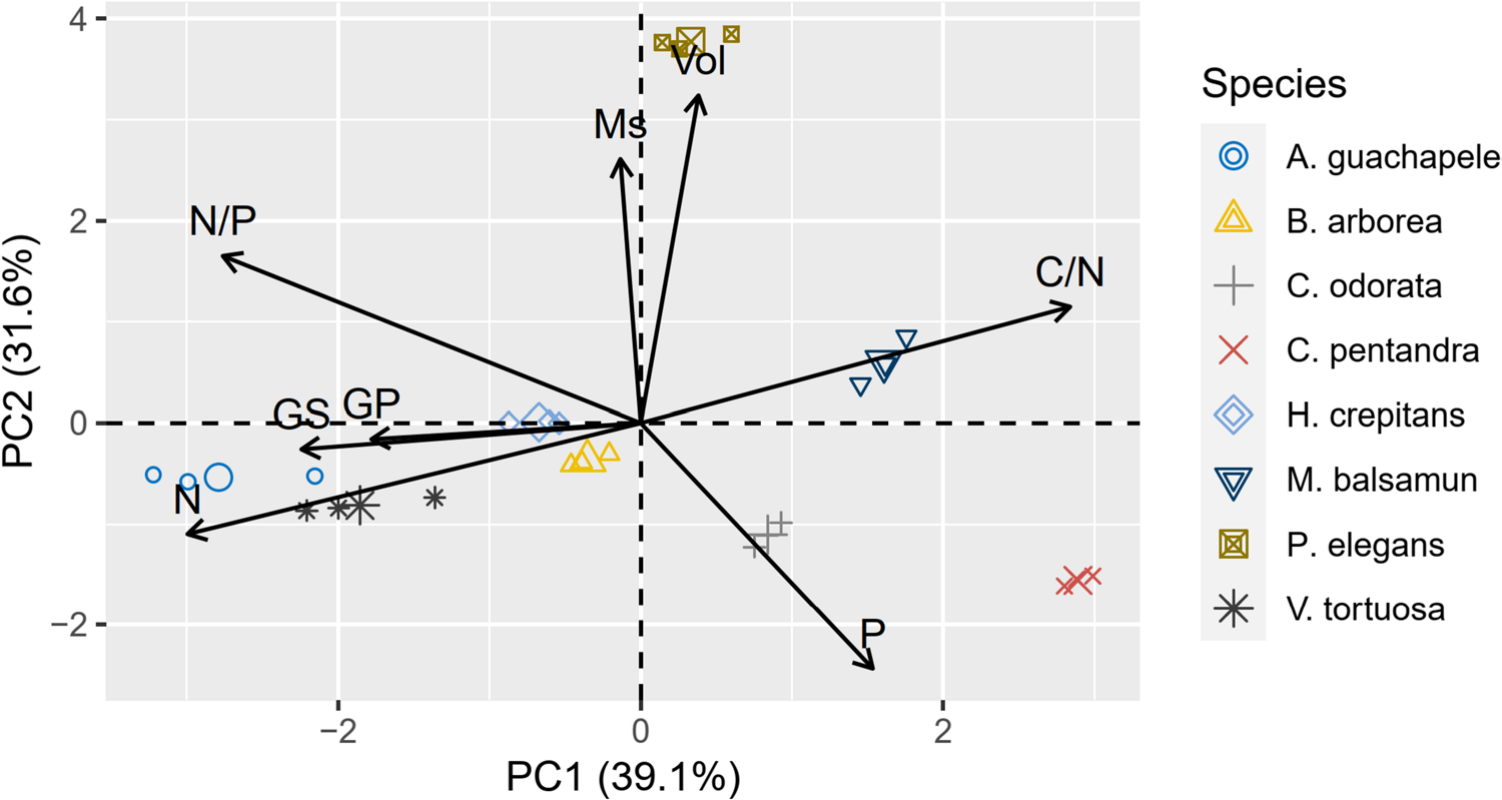
Principal component analysis (PCA) of functional traits and seed germination in eight TDF tree species of Magdalena, Colombia. Ms: mass, Vol: volume, C: carbon, N: nitrogen, P: phosphorus, *C*/*N*: carbon–nitrogen ratio, *N*/*P*: nitrogen-phosphorus ratio, GP: germination percentage, and GS: germination speed

Generally, seed traits showed a clear significant relationship with species germination (Fig. 4 and Table 2). In particular, the speed and percentage of germination (GS and GP) were positively related to mass (*r* = 0.52), N content (*r* = 0.41), and *N*/*P* ratio (0.70) and negatively with *C*/*N* ratio and *P* (*r* =  − 0.41). On the other hand, N and P contents were negatively associated with mass (Ms) and volume (Vol), respectively (Table 2). The results of this study indicated that 39.1% of the total variability was explained by the first principal component (PC1) and 31.6% by the second component (PC2) (Fig. 4). The first three PCA components explained 80.9% of the total variation in the data. The first component was associated with the variables, *C*/*N* (*r* =  − 0.52), GP (*r* = 0.29), N (*r* = 0.55), and GS (*r* = 0.37). The second component was associated with Ms (*r* =  − 0.47), *N*/*P* (*r* =  − 0.50), P (*r* = 0.44), and Vol (*r* =  − 0.54).

**Table 2:** Spearman’s correlations between functional traits, speed and average germination of eight TDF tree species of Magdalena, Colombia

	Mass	Volume	N	P	*N*/*P*	*C*/*N*	AG	GS
Mass		0.78***	− 0.18	− 0.53**	0.46*	0.13	0.52**	0.35
Volume			− 0.64**	− 0.43*	− 0.07	0.60**	0.07	− 0.12
N				0.26	0.47*	− 0.95***	0.46*	0.41*
P					− 0.54**	− 0.19	− 0.28	− 0.41*
*N*/*P*						− 0.58**	0.48*	0.70***
*C*/*N*							− 0.38	− 0.41*

*AG* average germination, *GS* germination speed

* Significant at 0.05 level; ** Significant at 0.01 level; *** Significant at 0.001 level

Two regression models were fitted and indicated the effect of some traits on seed germination of the species studied (Table 3). Although Model 1 was significant, it explained only 21% of the seed germination data (GP, *R*^2^). Specifically, *N* content was the most significant variable in this model. Specifically, Model 2 presented a better fit and was significant (*p* < 0.00; *R*^2^ = 42%). According to this model, the combined effect of volume and *N*/*P* ratio explained the speed of seed germination (Table 3).

**Table 3:** Regression models created using stepwise method of variable addition and elimination to explain the patterns between germination and functional traits in seeds of eight tree species in the TDFs of the department of Magdalena, Colombia

Model 1. Average germination	Variable	Added/removed	*R*^2^	AIC	RMSE
1	*N*	Added	20.80	26.72	0.39
Model 2. Germination speed					
1	*N*/*P*	Added	27.10	29.02	0.12
2	Vol	Added	42.00	32.49	0.11
Adjusted models		MSE	*R*^2^	DW	*p*-value
Model 1. GP = − 0.23 + 15.11N		0.15	20.80	1.80	0.03*
Model 2. GS = − 0.109 + 0.027NP − 0.0000216Vol		0.31	42.30	1.87	0.00***

*DW* Durbin-Watson, *MSE* mean squares of error, *AIC* Akaike information criterion, *RMSE* root mean squared error, *GP* germination percentage, *GS* germination speed

*Significant at 0.05 level; ***significant at 0.001

## Discussion

The present study reveals the importance of measuring functional traits to assess seed germination in several species found in TDF. Currently, there is limited information available (Visscher et al. 2022). Therefore, a better understanding of the functional characteristics of each species will enhance our knowledge of their biology, as well as their dynamics in natural regeneration and successional trajectories over time in these forests (Prado-Junior et al. 2017; Chaturvedi et al. 2021). Consequently, this advance not only enriches our scientific understanding but also allows the design of appropriate management strategies for the ecological conservation of these tropical dry ecosystems, which is a priority for their sustainability.

The results of this study partially supported our hypothesis, as not all seeds with greater mass and volume recorded high germination rates and percentages. On the contrary, these seeds showed lower nutrient contents (N and P) and more harder tissues (high *C*/*N* ratio, indicator of the quality of organic materials that may be resistant to decomposition in soil, e.g., tissues with high lignin, suberin, and polyphenol content) (Castellanos-Barliza and León-Peláez 2011; Gallagher et al. 2013). In this same sense, studies have indicated that hard-seedness may favor maintaining the embryo to prevent desiccation in long periods of dormancy in the seed bank, as well as protecting against mechanical impacts, insect attacks and pathogens until environmental conditions promote germination (Pacheco et al. 2007; Plaza and Magnitskiy 2007; Vargas-Figueroa 2015; Salvador et al. 2022).

Additionally, traits such as seed weight and volume have been associated with the dispersal ability, growth form and life history of the species (Leishman and Westoby 1994; Galindo-Rodriguez and Roa-Fuentes 2017; Chaturvedi et al. 2021). Similarly, patterns observed in TDF suggest that seeds with less weight (0.0016–0.8721 g) are a typical feature in the life cycles of early successional pioneer species such as *Eugenia procera* (Sw.) Poir., *Guazuma ulmifolia* Lam., and *Chiococca alba* (L.) Hitchc., which can disperse more easily and germinate quickly in open spaces (Otálora 2017; Prado-Junior et al. 2017). In our study, 88% (7 sp) of the species were intermediate pioneers, which presented generally low seed weights (≤ 1 g; according to Romero-Saritama and Pérez-Ruiz 2016), and 63% (5 sp) presented/had high germination percentages.

In this study, traits such as seed mass and seed volume did not predict germination speed or germination percentages, nor were they related to nutrient content (Fig. 4, Tables 2 and 3). Likely, seeds with greater mass and volume are more associated with dispersal processes and tolerance to dehydration (Galindo-Rodriguez and Roa-Fuentes 2017). In contrast, N content was the most important trait for the germination phase in this study (Table 3). These results are consistent with previous findings indicating that nutrient contents, particularly N and P, are more closely related to rapid seedling germination (Soriano et al. 2011; Veselá et al. 2022). However, most of the evaluated species are wind-dispersed and presented seeds with orthodox characteristics (≤ 1 g in weight), i.e., with hard and impermeable seed coats (Cárdenas-Salgado and Pizano 2019). *A. guachapele*, *H. crepitans*, and *V. tortuosa* species presented low *C*/*N* ratios and high N and P contents, suggesting fewer hard tissues and more N and P cycling. These high N contents favored high germination rates and percentages, particularly for *A. guachapele* and *V. tortuosa*. The higher nitrogen content could be attributed to their legumes' ability to associate with nitrogen-fixing bacteria. This association provides the advantage of accumulating more significant amounts of atmospheric nitrogen in their tissues, in the form of glycoproteins, amino acids, and tannins (Castellanos-Barliza and León-Peláez 2010; Corby et al. 2011; Soriano et al. 2015; Mathesius 2022). Similar results were observed by Soriano et al. (2011, 2015) in nine tree species of the Fabaceae family in a dry tropical forest in northwestern Mexico. High N contents (6.4–7.3%) in seeds were positively related to germination rates, attributed to the elevated mobilization of nitrogen compounds during the initial stages of germination.

In tropical forests, N and P are highly demanded elements in plant growth and development (Zhang et al. 2022). Therefore, during the early stages, large amounts of N are consumed in protein formation to generate rapid growth of stems and leaves (Nunes-Nesi et al. 2010). Likewise, P content is involved in root system development; therefore, larger P reserves in the seeds allow seedlings to be established faster (White and Veneklaas 2012; Novoa et al. 2018). We found that out of the eight species evaluated, *H. crepitans*, *M. balsamum,* and *B. arborea* had a low *N*/*P* ratio, which points to an adequate P content and likely an appropriate cycling of this element, which was reflected in high values in their germination rates (Fig. 2). On the other hand, *C. odorata* and *C. pentandra* did not germinate despite having a high N and P content; it is likely that these seeds quickly lost their viability when stored at room temperature or enter a stage of secondary dormancy (Pugnaire and Valladares 2007; Solberg et al. 2020), in which the seed is induced to enter a state of suspension of its development, remaining on standby until specific environmental factors such as the availability of water, light, temperature and soil moisture are conducive to the activation of the germination process (Baskin and Baskin 2004; Moles and Westoby 2006; Sautu et al. 2006; Garwood 1983; Rubio de Casas et al. 2017; Buijs 2020). Studies have reported that the probability of germination of *C. odorata* can vary between 10 and 70% due to specific requirements related to light availability and substrate temperature (Quinto et al. 2009; Alvarez and Rendón 2016). For *C. pentandra*, germination has been reported to occur between 18 and 41 days after sowing, with percentages ranging between 8 and 90%, depending on the time elapsed between harvest and sowing (Zamora-Cornelio et al. 2010).

Functional traits can generally predict processes in the dynamics of natural regeneration and seed germination in TDF ecosystems (Asanok et al. 2013; Faccion et al. 2021). However, the influence of these traits may vary according to the particular characteristics of each species and the ecosystem being evaluated. In this study, although N content was the best predictor of seed germination in all species (Table 3), the significant association observed between volume and *C*/*N* ratio (Table 2), reveals that seeds with higher volume presented more resistant tissues that retarded germination rates. Thus, functional traits such as the presence of thick testa and high values in the *C*/*N* ratio (hard tissues) could reveal the potential of some species to resist desiccation and persist in the seed bank until optimal conditions for their establishment as seedlings are present (Rahayu et al. 2022; Salvador et al. 2022). Consequently, these functional traits can determine a species’ reproductive success in the face of possible climate change and forest fire scenarios (Cárdenas-Salgado and Pizano 2019; Badano and Sánchez-Montes de Oca 2022).

## Conclusions

According to the observations of this study, the results did not clearly support our hypothesis, as larger seeds with higher mass and volume did not reflect higher nutrient contents favoring germination speed and germination percentages. On the other hand, functional traits such as nutrient content were more determinant seed mass and volume during the germination process, as in the case of *A. guachapele*, *B. arborea*, and *H. crepitans*. These species presented high N and P contents, resulting in their germination being high and fast. For this reason, evaluating several traits allows us to better assess the predictions and assumptions drawn from dynamic and vital processes like seed germination. The species *A. guachapele*, *B. arborea*, *H. crepitans*, and *V. tortuosa* demonstrated a high biological potential in terms of their regeneration capacity, particularly, because the characteristics of their seeds, as well as the nutrient content, revealed consistent implications in their reproductive success, promoting high germination percentages in less time. In general, the results obtained in this study provide basic knowledge for future research, offering starting points for further exploration of species-specific adaptations and how they may be affected by the environment. This more detailed focus on intraspecific variation in seed traits will contribute to our overall understanding of regeneration dynamics in tropical dry forests.
